# The Prognosis of Maintenance Hemodialysis Patients With Various Types of Vascular Access in Hemodialysis Centers in Wuhan: A Retrospective Cohort Study

**DOI:** 10.1155/ijne/5865205

**Published:** 2025-08-07

**Authors:** Li Cheng, Yonglong Min, Can Tu, Sheng Wan, Qianshen Zhu, Jing Chen, Wenhui Qiu, Nan Jiang, Hongbo Li

**Affiliations:** ^1^Department of Nephrology, Wuhan No.1 Hospital, Wuhan, China; ^2^Wuhan Blood Purification Clinical Research Center, Wuhan, China

**Keywords:** hemodialysis, prognosis, vascular access

## Abstract

**Objective:** The relationship between different types of vascular access in maintenance hemodialysis (MHD) patients and patient prognosis is controversial. The vascular access of patients from various dialysis centers in Wuhan was summarized, and its relationship with prognosis was analyzed.

**Methods:** The characteristics of MHD patients treated at 70 dialysis centers in the Wuhan Hemodialysis Quality Control System from 2017 to 2023 were collected. The demographic characteristics, laboratory indicators, compliance rates with laboratory indicators, annual mortality changes, survival time, and risk of death were compared in patients with various types of vascular access.

**Results:** A total of 45,830 MHD patients were included in the study. Overall, arteriovenous fistulas (AVFs) and tunneled and cuffed catheters (TCCs) remain the most common types of vascular access. Non-tunneled and cuffed catheters (NCC) use decreases annually, whereas arteriovenous graft (AVG) use increases annually. Male patients mostly had AVFs. The vascular access types of patients with diabetic nephropathy were mainly TCCs (28.6%) and AVGs (29.4%). AVG patients had the highest average hemoglobin level. NCC patients had the lowest average hemoglobin, albumin, and potassium levels. AVF patients had the highest average albumin, potassium, calcium, phosphorus, and parathyroid hormone levels. TCC patients had the lowest calcium and phosphorus levels. From 2017 to 2023, the mortality rates of AVF, TCC, and AVG patients were significantly higher in 2022 (11%, 19.9%, and 11.7%, respectively). The median survival time of AVF patients was 4.92 (2.75, 7.75) years, which was significantly longer than that of TCC patients (2.83 [1.42, 4.92]) and NCC patients (1.00 [0.25, 2.25]). After multivariate adjustment, the risk of death in patients with internal fistulas was 50.6% lower than that in patients with catheters, according to the Cox regression analysis model (hazard ratio = 0.494, 95% CI: 0.439–0.556, *p* < 0.001).

**Conclusions:** Among MHD patients with different vascular access types who were treated in Wuhan from 2017 to 2023, the numbers of AVF, AVG, and TCC patients increased with increasing overall number of MHD patients, whereas the number of NCC patients decreased. The overall condition and survival time of AVF patients were significantly better than those of MHD patients with other vascular access types, and the risk of death was lower.

## 1. Introduction

The number of maintenance hemodialysis (MHD) patients in China has been increasing annually. By the end of December 2023, a total of 916,647 patients had received MHD in mainland China, with 185,914 new MHD patients. The total prevalence of MHD is 635 per million people [1]. The effectiveness of MHD is directly related to the quality and stability of vascular access [2, 3]. Currently, the commonly used vascular access methods for MHD include autologous arteriovenous fistulas (AVFs), arteriovenous grafts (AVGs), tunneled and cuffed catheters (TCCs), and nontunneled and noncuffed catheters (NCCs).

The associations between various types of vascular access in MHD patients and patient prognosis are controversial. Most studies suggest that the mortality rate of MHD patients receiving AVFs is significantly lower than that of patients receiving catheters (including temporary catheters and long-term catheters) [4–6]. However, some studies suggest that vascular access-related complications can increase the risk of hospitalization in MHD patients but do not affect the prognosis of patients [2, 7]. Moreover, multicenter, large-sample, real-world cohort studies on the current situation and prognosis of vascular access in MHD patients in the central region of China are lacking. Therefore, the current situation of vascular access in 45,830 patients from 70 MHD centers in Wuhan and its relationship with prognosis were summarized and analyzed. We hope to provide a reference for the choice of vascular access in MHD patients.

## 2. Methods

### 2.1. Study Subjects

This retrospective cohort study was conducted between 2017 and 2023. The characteristics of MHD patients with complete vascular access-related information from 70 dialysis centers included in the Wuhan Hemodialysis Quality Control System (WHQCC) were collected. This study was approved by the Ethics Committee of the First Hospital of Wuhan (KY2022K0401) and registered at https://www.medicalresearch.org.cn (MR-42-24-024740). The trial was conducted in accordance with the Declaration of Helsinki (as revised in 2013). Adult MHD patients aged > 18 years with a regular hemodialysis duration of more than three months were included. MHD patients with > 20% missing data were excluded. Patients with acute kidney injury whose temporary catheters were removed after recovery from dialysis were also excluded. Patients who refused to connect or were lost to follow-up were also excluded. All patients signed an informed consent form.

### 2.2. Data Collection

Patient demographic characteristics, including hospital level, age, sex, dialysis duration, and primary disease, were collected through the WHQCC. The type of vascular access and laboratory indicators, including hemoglobin, parathyroid hormone, serum albumin, potassium, calcium, phosphorus, parathyroid hormone, and serum ferritin levels, were collected from the WHQCC. Changes in the mortality rate of MHD patients with different vascular access routes from 2017 to 2023 were observed, and the overall survival rates of MHD patients with different vascular access routes were compared. Overall survival was defined as the time from the initial stage of dialysis to withdrawal or death. MHD patients may withdraw due to kidney transplantation, treatment cessation, or other reasons. Finally, the Cox regression analysis model was used to analyze the risk of death in patients with different vascular access types.

### 2.3. Statistical Methods

SPSS 22.0 software was used for the statistical processing of the data. Measurement data that conformed to a normal distribution are reported as (*x* ± *s*), measurement data that did not conform to a normal distribution are reported as M (P25, P75), and count data are presented as *n* (%). Analysis of variance was used for comparisons between multiple groups of normally distributed data, the Mann‒Whitney U test was used for comparisons between two groups of data with a skewed distribution, the Kruskal‒Wallis test was used for comparisons between multiple groups, and the *χ*^2^ test was used for comparisons of count data. The Kaplan‒Meier survival curve (log-rank test) method was used to compare the differences in survival between the two groups. *p* < 0.05 was considered statistically significant. Cox proportional hazards regression models were used to analyze the impacts of different vascular access types on mortality risk in MHD patients.

## 3. Results

### 3.1. Changes in Vascular Access

**Figure 1:**
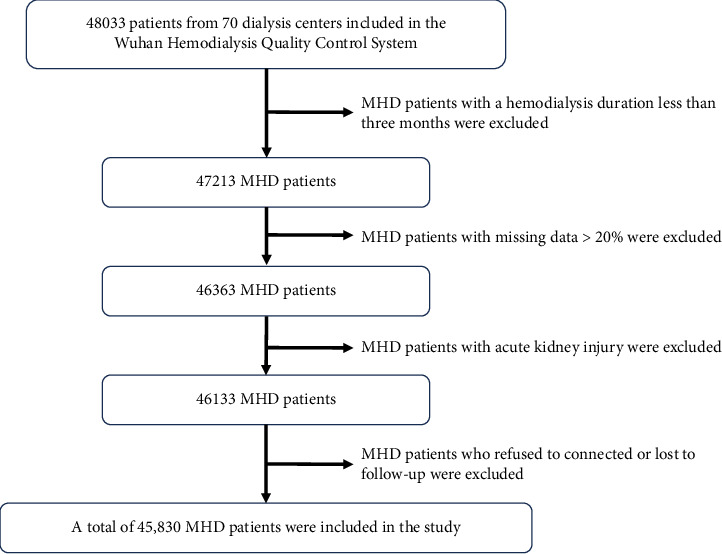
The screening process for eligible patients included in the study.

**Figure 2:**
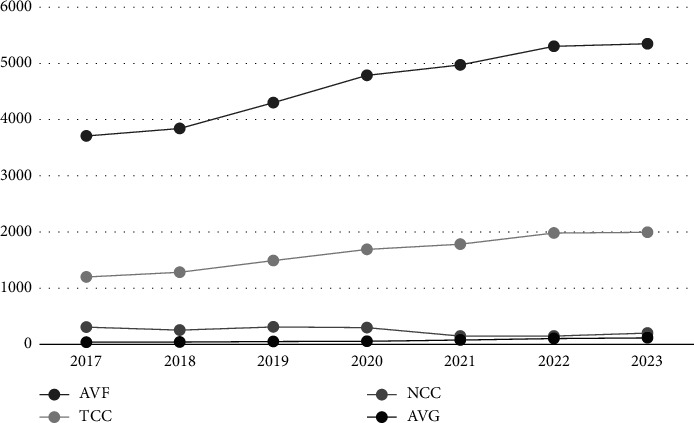
Changes in vascular access in the Wuhan Hemodialysis Quality Control System from 2017 to 2023.

A total of 45,830 MHD patients were included in the study (Figure 1). From 2017 to 2023, AVF was the most commonly used vascular access type in hemodialysis patients (69.8%–71.2%), and the percentages of TCCs and NCCs used were 22.8%–26.3% and 2.0%–5.8%, respectively. AVF and TCC use showed a significant increasing trend, and the percentage of NCC use decreased annually. Although the percentage of AVG use was the lowest (0.7%–1.5%), it showed an increasing trend (Figure 2). This trend may be related to the limited promotion of the AVG establishment technique and economic reasons.

### 3.2. Comparison of the Demographic Characteristics of MHD Patients With Different Vascular Access Types

Significant differences in the type of dialysis center, sex, age, dialysis duration, and primary disease were observed among MHD patients with different vascular access types. In this study, most of the dialysis centers were tertiary medical institutions (accounting for 81.3%). With improvements at the hospital level, the percentages of AVF and AVG use gradually increased, whereas the percentages of TCC and NCC use gradually decreased. Among the MHD patients, 61.9% were male, and 38.1% were female. Male patients were more likely to choose AVF, whereas female patients were more likely to choose TCC and NCC. The average age of the TCC patients was the oldest (69.27 ± 13.84 years), and the average age of the AVF patients was the youngest (63.57 ± 13.69 years). The dialysis duration of the AVF patients was 4.08 (2.17, 6.83) years, the dialysis duration of the TCC patients was 4.75 (2.67, 7.50) years, the dialysis duration of the NCC patients was 2.83 (1.50, 4.83) years, and the dialysis duration of the AVG patients was 1.25 years (0.50, 2.42). Among all patients with vascular access, secondary glomerular disease was the most common primary condition, followed by primary glomerular disease (Table 1).

### 3.3. Comparison of the Laboratory Indicators of MHD Patients With Different Vascular Access Types

Significant differences in hemoglobin, platelet, albumin, potassium, calcium, phosphorus, parathyroid hormone, and serum iron levels were observed among patients with different vascular access types. AVG patients had the highest average hemoglobin level and the lowest platelet, parathyroid hormone, and serum iron contents. NCC patients had the lowest average hemoglobin, albumin, and potassium levels but the highest platelet and serum iron contents. AVF patients had the highest average albumin, potassium, calcium, phosphorus, and parathyroid hormone levels. TCC patients had the lowest calcium and phosphorus levels (Table 2).

### 3.4. Comparison of the Compliance Rates of Laboratory Indicators in MHD Patients With Different Vascular Access Types

Furthermore, MHD patients with different vascular access types were grouped and analyzed according to whether the biochemical indicators met the standards. The results showed that AVF patients had higher compliance rates for hemoglobin, albumin, serum calcium, and parathyroid hormone levels. NCC patients had higher compliance rates for potassium and serum iron levels. TCC patients had higher compliance rates for phosphorus levels (Table 3).

### 3.5. Changes in the Mortality Rates of MHD Patients With Different Vascular Access Types

**Figure 3:**
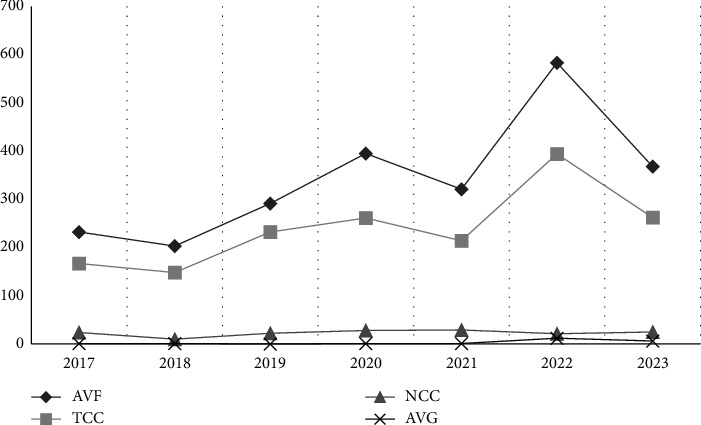
Changes in the mortality rates of MHD patients with different types of vascular access.

The mortality rates of patients with different vascular access types varied from 2017 to 2023. A significant difference in the mortality rate of AVF patients was observed between different years. In 2022, the mortality rates of MHD patients with vascular access through AVFs, TCCs, and AVGs were significantly higher (11%, 19.9%, and 11.7%, respectively) than those reported in previous years. In 2019, the mortality rate of MHD patients with vascular access through NCCs was significantly greater (19.6%) (Figure 3).

### 3.6. Comparison of the Survival Time of MHD Patients With Different Vascular Access Types

**Figure 4:**
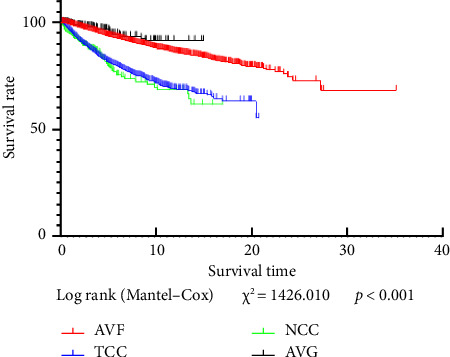
Comparison of the survival times of MHD patients with different types of vascular access.

The median survival times of patients with AVF, TCC, NCC, and AVG were 4.92 (2.75, 7.75) years, 2.83 (1.42, 4.92) years, 1.00 (0.25, 2.25), and 5.00 (2.50, 7.83) years, respectively. The survival times of AVF and AVG patients were significantly longer than those of TCC and NCC patients (log rank (Mantel‒Cox) *χ*^2^ = 1426.010, *p* < 0.001) (Figure 4).

### 3.7. Cox Regression Analysis of the Effects of Different Vascular Access Types on the Risk of Death in MHD Patients

Vascular access was divided into internal fistulas (including AVFs and AVGs) and catheters (including TCCs and NCCs). Without adjusting for confounding factors, the risk of death in patients with internal fistulas as the type of vascular access was 67.5% lower than that in patients with catheters (HR = 0.325, 95% CI: 0.306–0.346; *p* < 0.001). With the continuous increase in adjusted variables in the model, the HR increased slightly. The Cox regression model after multivariate adjustment revealed that the risk of death in patients with internal fistulas as the vascular access type was 50.6% lower than that in patients with catheters (HR = 0.494, 95% CI: 0.439–0.556, *p* < 0.001) (Table 4).

## 4. Discussion

The results of this study revealed that as time progressed, changes in the types of vascular access occurred in MHD patients in Wuhan. Among them, the numbers of AVF, AVG, and TCC patients tended to increase, whereas the number of NCC patients tended to decrease. Although this study included 70 dialysis centers in Wuhan and differences in management philosophies, staff compositions, or environmental factors exist among different dialysis centers, the vascular access of MHD patients still aligns with this trend. We speculate that patients' understanding of kidney disease and dialysis is gradually increasing in the field of chronic kidney disease. Therefore, the number of patients who progress to end-stage renal disease (ESRD) and require NCC for emergency dialysis is gradually decreasing. An increasing number of patients recognize the importance of preparing vascular access for MHD in advance. This finding is consistent with the study by Kukhon et al. [8]. Moreover, Chen et al. [9] found that patients who had a mature AVF or AVG at the initial dialysis session had significantly lower anxiety levels than those with NCCs or TCCs. Preparing vascular access for MHD in advance can help MHD patients relieve their anxiety during the initial dialysis session. However, this recommendation is still influenced by other factors, such as prolonged referral times (resulting in a delayed surgical evaluation for vascular access), a shortage of vascular access surgeons, or decisions made by the directors of public hospitals [6]. Therefore, in practice, this choice is still influenced by factors such as environmental conditions and policies. On the other hand, with the gradual maturity of vascular access technology, AVG is more widely used in MHD patients whose vascular conditions do not meet the requirements for AVF in Wuhan [10, 11]. The application of AVGs instead of TCCs in MHD patients has also been shown to significantly increase the survival rate of patients with ESRD and reduce the risk of infection, hospitalization, and medical expenses [12].

Moreover, this study revealed significant differences in the general characteristics, laboratory indicators, and compliance rates of laboratory indicators among MHD patients with different vascular access types. More AVF patients were young males, whereas more TCC patients were elderly females. We speculated that the choice of vascular access is related to the vascular conditions of MHD patients. Male patients have better vascular conditions and are more inclined to choose AVF. Elderly female patients have vascular conditions that are not conducive to the maturation or use of AVFs and are thus more inclined to choose TCCs. Furthermore, regarding the etiological distribution of primary diseases, while primary glomerular diseases historically account for most ESRD cases, the proportion of ESRD cases caused by secondary glomerular diseases (including hypertensive nephropathy, diabetic nephropathy, and hyperuricemia-related nephropathy) has steadily increased with socioeconomic development [13]. In terms of laboratory indicators and compliance rates, AVF patients were significantly better than patients with other types of vascular access, which is also consistent with the findings of previous studies [6]. These findings suggest that the dialysis adequacy, nutritional status, and survival status of AVF patients are significantly better than those of patients with other types of vascular access. Consistently, this study revealed that the survival time of AVF patients was significantly longer than that of MHD patients with other vascular access types. The elevated mortality in NCC patients during the COVID-19 pandemic may be linked to an increase in acute kidney injury cases necessitating intubation and dialysis [14]. More than 100,000 publications related to COVID-19 are available in PubMed, including more than 800 concerning both COVID-19 and acute kidney injury [15]. SARS-CoV-2 can infect not only renal podocytes and proximal tubular cells via angiotensin-converting enzyme 2 (ACE2), leading to acute tubular necrosis, protein leakage from Bowman's capsule, collapsing glomerulopathy, and mitochondrial damage but also immune dysregulation, including a cytokine storm, macrophage activation syndrome, and lymphopenia [16]. All of these factors can contribute to acute kidney injury. In patients with severe disease, intubation and dialysis may be needed. Moreover, when the Cox regression analysis model was used to analyze the impacts of vascular access type on death, the risk of death in patients with internal fistulas as the type of vascular access was lower than that in patients with catheters. This finding is consistent with those of previous studies. A retrospective survey from the United States including 400,000 patients revealed that the initial use of catheters instead of AVFs in MHD patients was associated with a 51% increase in mortality [17]. Moreover, the long-term application of TCC was associated with a 2.2-fold increase in mortality. The infections and mortality associated with TCC worsen with age [17, 18]. However, Professor Fu Ping from West China Hospital and others reported that AVFs or TCCs were not associated with mortality in a 25.8 (12–43)-month follow-up of 358 elderly MHD patients. In contrast, diastolic blood pressure and left heart failure were found to be independent influencing factors for CVD-related death. The latest “Clinical Practice Guideline for Vascular Access” released by the KDOQI emphasizes the “patient-first” principle and has been proposed for use in clinical practice. Thus, individualized vascular access should be selected after factors such as the patient's life expectancy, personal preferences, and medical conditions are considered [2].

This study has the following limitations. First, the cohort study did not account for the impacts of socioeconomic factors on the prognosis of MHD patients. Second, MHD patients may have comorbidities such as cerebrovascular disease, cardiovascular abnormalities, and arrhythmias, which could also influence the outcomes of MHD patients with different vascular access types. Third, the findings still require further validation through prospective cohort studies.

In summary, our study revealed that the number of MHD patients with AVFs, AVGs, and TCCs in Wuhan from 2017 to 2023 tended to increase with increasing overall number of MHD patients, whereas the number of MHD patients with NCCs tended to decrease. The overall status and survival time of AVF patients were significantly better than those of MHD patients with other types of vascular access. The risk of death of AVF patients was lower.

## Figures and Tables

**Table 1:** Comparison of the demographic characteristics of MHD patients with different vascular access types.

		**N**	**AVF**	**TCC**	**NCC**	**AVG**	*χ* ^2^ **/F**	**p**

Hospital level	Hospital without a defined grade	502 (1.1)	387 (1.2)	104 (0.9)	6 (0.4)	5 (1)	297.482	< 0.001^λ^
	Primary-level hospital	1815 (4)	1533 (4.8)	263 (2.3)	9 (0.5)	10 (2.1)		
	Primary-level hospital	6245 (13.6)	4554 (14.1)	1523 (13.3)	135 (8.1)	33 (6.8)		
	Tertiary-level hospital	37268 (81.3)	25782 (79.9)	9531 (83.5)	1520 (91)	435 (90.1)		

Gender	Male	28372 (61.9)	21452 (66.5)	5678 (49.7)	976 (58.4)	266 (55.1)	1027.142	< 0.001^λ^
	Female	17458 (38.1)	10804 (33.5)	5743 (50.3)	694 (41.6)	217 (44.9)		

Age (years)		63.57 ± 13.69	61.51 ± 13.03	69.27 ± 13.84	64.24 ± 14.55	63.94 ± 11.28	957.775	< 0.001^γ^

Dialysis age (years)		4.08 (2.17 , 6.83)	4.75 (2.67, 7.50)	2.83 (1.50, 4.83)	1.25 (0.50, 2.42)	4.58 (2.31, 7.83)	4863.896	< 0.001^β^

Primary disease	Primary glomerular disease	14994 (33.3)	11634 (36.4)	2983 (26.6)	240 (17.7)	137 (28.4)	7295.911	< 0.001^λ^
	Secondary glomerular disease	21657 (48.1)	14917 (46.6)	6146 (54.9)	340 (25.0)	254 (52.6)		
	Hereditary or congenital nephropathy	1034 (2.3)	831 (2.6)	195 (1.7)	1 (0.1)	7 (1.4)		
	Tubulointerstitial kidney disease	292 (0.6)	199 (0.6)	73 (0.7)	13 (1.0)	7 (1.4)		
	Urinary tract tumors	107 (0.2)	44 (0.1)	54 (0.5)	3 (0.2)	6 (1.2)		
	Urinary tract infections and stones	581 (1.3)	420 (1.3)	148 (1.3)	4 (0.3)	9 (1.9)		
	Post-nephrectomy	48 (0.1)	28 (0.1)	18 (0.2)	2 (0.1)	0		
	Acute renal failure	53 (0.1)	36 (0.1)	12 (0.1)	5 (0.4)	0		
	Renal transplant dysfunction	245 (0.5)	169 (0.5)	58 (0.5)	4 (0.3)	14 (2.9)		
	Unknown	6028 (13.4)	3715 (11.6)	1518 (13.6)	746 (44.9)	49 (10.1)		

*Note:* Categorical variables were expressed as number (%). *p* values were calculated by *χ*^2^ test (λ). Continuous variables were presented as medium (P25, P75) or mean and standard deviation (SD). *p* values were calculated by analysis of variance for normal distributed data (γ) or Kruskal–Wallis test for skewed distributed data (β).

Abbreviation: AVG, arteriovenous graft; NCC, nontunnel and cuffed catheter; TCC, tunneled and cuffed catheter.

**Table 2:** Comparison of the laboratory indicators of MHD patients with different vascular access types.

	*N*	AVF	TCC	NCC	AVG	*χ* ^2^/*F*	*p*
Haemoglobin (g/L)	105.37 ± 19.24 *n* = 38192	106.94 ± 18.69 *n* = 27414	101.94 ± 19.79^a^ *n* = 9305	93.71 ± 21.17^a,b^ *n* = 1043	108.34 ± 17.38^a,b,c^ *n* = 430	297.083	< 0.001^γ^
Blood platelet (g/L)	171.48 ± 64.82 *n* = 34470	171.83 ± 62.59 *n* = 24563	169.06 ± 68.93^a^ *n* = 8471	186.84 ± 78.10^a,b^ *n* = 1023	162.67 ± 65.82^a,c^ *n* = 413	25.929	< 0.001^γ^
Serum albumin (mmol/L)	38.758 ± 4.52 *n* = 35217	39.326 ± 4.22 *n* = 25277	37.435 ± 4.86^a^ *n* = 8628	35.567 ± 5.76^a,b^ *n* = 909	38.664 ± 3.51^a,b,c^ *n* = 403	554.83	< 0.001^γ^
Potassium (mmol/L)	4.73 ± 0.84 *n* = 34396	4.794 ± 0.83 *n* = 24573	4.591 ± 0.87^a^ *n* = 8433	4.476 ± 0.90^a,b^ *n* = 990	4.699 ± 0.83^a,b,c^ *n* = 400	154.658	< 0.001^γ^
Calcium (pm/mL)	2.22 ± 0.24 *n* = 37601	2.23 ± 0.24 *n* = 27072	2.19 ± 0.23^a^ *n* = 9104	2.14 ± 0.23^a,b^ *n* = 1003	2.21 ± 0.22^c^ *n* = 422	85.850	< 0.001^γ^
Phosphorus (μg/L)	1.76 ± 0.60 *n* = 37133	1.81 ± 0.59 *n* = 26784	1.62 ± 0.59^a^ *n* = 8918	1.62 ± 0.60^a^ *n* = 1010	1.70 ± 0.57^a,b,c^ *n* = 421	258.354	< 0.001^γ^
Parathyroid hormone (pm/mL)	286.70 (153.65, 526.30) *n* = 34046	305.30 (168.16, 554.18) *n* = 24859	241.70 (122.50, 458.40)^a^ *n* = 8001	231.45 (117.98, 407.85)^a^ *n* = 796	242.01 (117.95, 453.00)^a^ *n* = 390	389.075	< 0.001^β^
Serum ferritin (μg/L)	138.90 (54.89, 304.93) *n* = 25476	139.90 (55.91, 305.150) *n* = 18289	131.5 (49.84, 296.90)^a^ *n* = 6240	193.40 (80.71, 379.58)^a,b^ *n* = 649	137.13 (47.85, 336.66) *n* = 328	40.737	< 0.001^β^

*Note:* Continuous variables were presented as medium (P25, P75) or mean and standard deviation (SD). *p* values were calculated by analysis of variance for normal distributed data (γ) or Kruskal–Wallis test for skewed distributed data (β). TCC, tunneled and cuffed catheter.

Abbreviation: AVG, arteriovenous graft; NCC, nontunnel and cuffed catheter; TCC, tunneled and cuffed catheter.

^a^compared with AVF, *p* < 0.05.

^b^compared with TCC, *p* < 0.05.

^c^compared with NCC, *p* < 0.05.

**Table 3:** Comparison of the compliance rates of laboratory indicators in MHD patients with different vascular access types.

		**N**	**AVF**	**TCC**	**NCC**	**AVG**	*χ* ^2^	**p**

Haemoglobin	< 110	21384 (56)	14579 (53.2)	5818 (62.5)	770 (73.8)	217 (50.5)	397.526	< 0.001^λ^
	110–130	13744 (36)	10432 (38.1)	2899 (31.2)	234 (22.4)	179 (41.6)		
	> 130	3064 (8)	2403 (8.7)	588 (6.3)	39 (3.7)	34 (7.9)		

Serum albumin	< 40	20253 (57.5)	13444 (53.2)	5863 (68)	693 (76.2)	253 (62.8)	713.459	< 0.001^λ^
	≥ 40	14964 (42.5)	11833 (46.8)	2765 (32)	216 (23.8)	150 (37.2)		

Serum potassium	≤ 5.0	22395 (65.1)	15409 (62.7)	5977 (70.9)	731 (73.8)	278 (69.5)	222.486	< 0.001^λ^
	> 5.0	12001 (34.9)	9164 (37.3)	2456 (29.1)	259 (26.2)	122 (30.5)		

Serum calcium	< 2.1	10934 (29.1)	7423 (27.4)	2946 (32.4)	425 (42.4)	140 (33.2)	227.946	< 0.001^λ^
	2.1–2.5	22785 (60.6)	16599 (61.3)	5423 (59.6)	519 (51.7)	244 (57.8)		
	> 2.5	3882 (10.3)	3050 (11.3)	735 (8.1)	59 (5.9)	38 (9)		

Serum phosphorus	< 1.13	4838 (13)	2845 (10.6)	1731 (19.4)	193 (19.1)	69 (16.4)	799.415	< 0.001^λ^
	1.13–1.78	15787 (42.5)	11006 (41.1)	4141 (46.4)	462 (45.7)	178 (42.3)		
	> 1.78	16508 (44.5)	12933 (48.3)	3046 (34.2)	355 (35.1)	174 (41.3)		

Parathyroid hormone	< 150	8489 (24.9)	5570 (22.4)	2547 (31.8)	248 (31.2)	124 (31.8)	362.137	< 0.001^λ^
	150–600	18443 (54.2)	13708 (55.1)	4106 (51.3)	432 (54.3)	197 (50.5)		
	> 600	7114 (20.9)	5581 (22.5)	1348 (16.8)	116 (14.6)	69 (17.7)		

Serum ferritin	< 200	15346 (60.2)	10994 (60.1)	3828 (61.6)	332 (51.2)	192 (58.5)	30.509	< 0.001^λ^
	200–500	6784 (26.6)	4905 (26.8)	1580 (25.4)	215 (33.1)	84 (25.6)		
	> 500	3346 (13.1)	2390 (13.1)	802 (12.9)	102 (15.7)	52 (15.9)		

*Note:* Categorical variables were expressed as number (%). *p* values were calculated by *χ*^2^ test (λ).

Abbreviations: AVG, arteriovenous graft; NCC, nontunnel and cuffed catheter; TCC, tunneled and cuffed catheter.

**Table 4:** Cox regression analysis of the impacts of different types of vascular access on the risk of death in MHD patients.

Model	AVF as vascular access		
	HR	95% CI	*p*
Unadjusted confounding	0.325	0.306–0.346	< 0.001
Model 1	0.448	0.419–0.478	< 0.001
Model 2	0.453	0.424–0.483	< 0.001
Model 3	0.494	0.439–0.556	< 0.001

*Note:* Model 1: general characteristics including hospital level, gender, age, and primary diseases, etc. Model 2: including Model 1 and dialysis modalities. Model 3: including Model 2 and laboratory results such as hemoglobin, platelets, albumin, potassium, etc. In the models, the analysis was carried out with the vascular access as the catheter as a reference.

## Data Availability

The data that support the findings of this study are available from the corresponding author upon reasonable request.
